# Broad induction of immunoregulatory mechanisms after a short course of anti-IL-7Rα antibodies in NOD mice

**DOI:** 10.1186/s12865-017-0201-4

**Published:** 2017-03-29

**Authors:** Cristina Vazquez-Mateo, Justin Collins, Michelle Fleury, Hans Dooms

**Affiliations:** Department of Medicine, Arthritis Center/Rheumatology Section, Boston University School of Medicine, 72 East Concord Street, E519, Boston, MA 02118 USA

**Keywords:** Type 1 diabetes, Interleukin 7, T cells, Autoimmunity, Tregs, Inhibitory receptors, Non-obese diabetic mice

## Abstract

**Background:**

Type 1 diabetes is an autoimmune disease caused by T cell-mediated destruction of the insulin-producing β-cells in the pancreas. Therefore, approaches that effectively halt the pathogenic T cell response are predicted to have preventive or therapeutic benefit for type 1 diabetes patients. We previously demonstrated that long-term blocking of IL-7 signaling, which is critical for the survival and function of T cells, prevented and reversed type 1 diabetes in non-obese diabetic mice. However, such persistent inhibition of T cell responses raises concerns about causing immunodeficiency. Here, we asked whether a reduced duration of the treatment with anti-IL-7Rα antibodies retained efficacy in preventing diabetes. Moreover, we sought to identify immunoregulatory mechanisms induced by anti-IL-7Rα administration.

**Results:**

Anti-IL-7Rα antibodies were administered to prediabetic NOD mice for 3 weeks and blood samples were taken at the end of treatment and 2 weeks later to analyze changes in T cell phenotypes in response to IL-7Rα blockade. We found that the co-inhibitory receptors LAG-3, Tim-3 and PD-1 were increased on peripheral blood CD4^+^ and CD8^+^ T cells from anti-IL-7Rα-treated mice. Expression of these receptors contributed to reduced T cell cytokine production in response to TCR stimulation. In addition, the frequency of Tregs within the circulating CD4^+^ T cells was increased at the end of anti-IL-7Rα antibody treatment and these Tregs showed a more activated phenotype. In vitro restimulation assays revealed that effector T cells from anti-IL-7Rα-treated mice were more sensitive to co-inhibitory receptor induction after TCR stimulation. Importantly, these changes were accompanied by delayed type 1 diabetes disease kinetics.

**Conclusions:**

Together, our data show that short-term blockade of IL-7Rα induces detectable changes in co-inhibitory receptor expression and Treg frequencies in peripheral blood of NOD mice. These changes appear to have long-lasting effects by delaying or preventing type 1 diabetes incidence. Hence, our study provides further support for using anti-IL-7Rα antibodies to modulate autoreactive T cell responses.

## Background

Type 1 diabetes is a progressive autoimmune disease caused by infiltration of autoreactive lymphocytes in the islets of Langerhans which, ultimately, will destroy the insulin-producing β-cells. As a result of the loss of β-cells, blood sugar levels increase leading to a severe risk of secondary organ complications. Despite current advances in the understanding of type 1 diabetes, treatment remains largely limited to insulin replacement therapy and attempts to prevent or cure the disease in humans have so far been unsuccessful [1, 2].

IL-7 is a cytokine with an important role in T cell survival and function and is an emerging target for the treatment of multiple autoimmune diseases [3]. We and others previously demonstrated that blocking IL-7 receptor alpha (IL-7Rα) prevented and reversed diabetes in non-obese diabetic (NOD) mice and hence has potential to be translated as an immunotherapy for human type 1 diabetes [4, 5]. Initial analyses of CD4^+^ T cells in anti-IL-7Rα-treated mice revealed increased expression of the co-inhibitory receptor Programmed Death-1 (PD-1) in effector/memory CD4^+^ T cells (T_E/M_) and an increased frequency of polyclonal regulatory T cells (Tregs) in lymphoid organs [4]. These observations suggested that anti-IL-7Rα antibodies shift the balance in the immune system from active autoreactivity to a more regulated state, impacting disease progression.

Co-inhibitory receptors play critical roles in maintaining self-tolerance to autoantigens and are also associated with “T cell exhaustion”, caused by chronic antigenic stimulation of virus- and tumor-specific T_E/M_ cells [6–8]. Hence, increasing co-inhibitory receptor expression and “exhaustion” in autoreactive T cells are predicted to be desirable outcomes for the treatment of autoimmune diseases such as type 1 diabetes. Loss-of-function studies of the co-inhibitory receptors PD-1 and LAG-3 have demonstrated a critical role for these co-inhibitory receptors in suppressing anti-islet T cell responses in NOD mice, reflected by an accelerated kinetics of disease course [9–12]. Contributions of other co-inhibitory receptors, e.g., Tim-3 and B7x, in regulating type 1 diabetes are emerging as well [13, 14]. The role of Tregs in maintaining islet tolerance is also firmly established and defects in Tregs may underlie susceptibility for type 1 diabetes [15, 16]. Various approaches to increase Treg activity for the treatment of type 1 diabetes are intensively being developed and, in some cases, have entered clinical trials [17].

The initiation of clinical trials to use anti-IL-7Rα antibodies for the treatment of type 1 diabetes and other autoimmune diseases [18] underscores the necessity to better understand the treatment modalities and mechanisms underlying protection against type 1 diabetes provided by anti-IL-7Rα administration. Therefore, we treated prediabetic NOD mice with a short course of anti-IL-7Rα antibodies and expanded our analysis of co-inhibitory receptor expression and Tregs. We found that in addition to PD-1, LAG-3 and Tim-3 were also induced on T cells in response to IL-7Rα blockade. Importantly, changes in these receptors could be found not only in lymphoid organs but on peripheral blood T cells as well and may serve as a biomarker of treatment efficacy. Furthermore, we show that IL-7Rα blockade increases the frequency and changes the phenotype of polyclonal Tregs. Together, our data suggest that anti-IL-7Rα antibodies promote two key mechanisms of protection against autoimmunity: increased expression of co-inhibitory receptors and increased Treg activity. Moreover, short-term IL-7Rα blockade retained some capacity to alter the kinetics of the disease.

## Methods

### Mice

Prediabetic female NOD mice (9–11 weeks) were purchased from The Jackson Laboratory (US). All animal experiments were approved by the Institutional Animal Care and Use Committee of Boston University Medical Campus.

### Diabetes assessment

Diabetes incidence was followed weekly by urine analysis (Diastix, Bayer, US) and measuring of blood glucose levels with a Contour glucose meter (Bayer; US). The percentage of diabetic mice (glucose levels >250 mg/dL) over a 32-weeks time course was calculated by the Survival Curves method using GraphPad Prism.

### *In vivo* antibody treatment

Anti-IL-7Rα (rat IgG2a, clone A7R34) antibodies for in vivo blocking experiments were produced by a hybridoma cell line and purified with Protein G Sepharose 4 Fast Flow (GE Healthcare, US) in our laboratory. Rat IgG (Jackson ImmunoResearch Laboratories, US) was used as a control. For anti-IL-7Rα and rat IgG antibodies, 0.5 mg was administered in PBS intraperitoneally.

### *In vitro* stimulation assays and ELISA

Cells were cultured in RPMI 1640 media (Invitrogen, US) supplemented with 1 mM each of L-glutamine, nonessential amino acids, sodium pyruvate, Hepes, penicillin, streptomycin, 50 μM 2-Mercaptoethanol (Gibco by Life Technologies, US), and 10% FCS (Omega Scientific, US), and incubated at 37 °C in 5% CO2. In vitro assays to measure cytokine production were performed by stimulating 5×10^5^ cells from spleen and pancreatic lymph nodes (PLN) with soluble anti-CD3 (1 μg/ml) (clone 145-2C11; eBioscience, US) and anti-CD28 (2 μg/ml) (clone 37.51; eBioscience, US) antibodies in round-bottom 96-well plates (BD Falcon, US) in the absence or presence of blocking antibodies (10 μg/ml) for PD-L1 (clone MIH5), LAG-3 (clone C9B7W) and Tim-3 (clone RMT3-23) (Bio X Cell, US). Supernatants from the cultures were harvested after 18 h and IFN-γ and IL-2 content determined by ELISA (eBioscience, US), following the manufacturer’s instructions. For assays to measure induction of co-inhibitory receptor expression, PLN cells from mice treated with anti-IL-7Rα or rat IgG antibodies were stimulated in vitro with soluble anti-CD3- (0.1 or 10 μg/ml) and anti-CD28 (1 μg/ml) antibodies. Cell cultures were set up in flat-bottom 96-well plates (BD Falcon, US) and harvested after 3 days for flow cytometric analysis.

### Antibodies and staining procedures

Blood samples (50–100 μl) were drawn from mouse tail vein and an equal volume of EDTA (50 mM) (Sigma, US) was added immediately to avoid coagulation. Prior to staining, erythrocytes were lysed for spleen and blood samples. To distinguish live from dead cells, cells were preincubated with a fixable viability dye (eBioscience, US) according to manufacturer’s instructions. Fc receptors were blocked with anti-CD16/CD32 antibodies for 5 min at 4 °C before any antibody staining procedures were started. The following antibodies were used for detection of murine activation and proliferation markers and co-inhibitory receptors: anti-CD4; anti-CD8; anti-PD-1; anti-Tim-3; anti-LAG-3; anti-CD44; anti-Foxp3 and anti-CD25 (eBioscience, Biolegend or BD Pharmingen, US). Extracellular staining was performed by incubating with antibodies for 15–30 min at 4 ° C. For Foxp3 intracellular staining cells were fixed and permeabilized with a Foxp3 staining buffer set (eBioscience, US) following manufacturer’s instructions.

### Flow cytometry

Phenotypic analysis of cell populations was performed by multiparameter flow cytometry. Fluorescence intensities were measured on a LSRII flow cytometer and data were analyzed with FlowJo software.

### Statistics

Statistically significant differences between groups were determined using the Mantel–Cox log-rank test (for diabetes incidence) and one- or two-tailed paired or unpaired t tests (for flow cytometry data) using Graph Pad Prism. *P* values ≤ 0.05 were considered significant. Horizontal lines in graphs indicate statistical significance (* = *p* ≤ 0.05, ** = *p* ≤ 0.005, *** = *p* ≤ 0.0005, ns = *p* > 0.05).

## Results

### A short course of IL-7Rα blocking antibodies delays type 1 diabetes onset in NOD mice

**Fig. 1:**
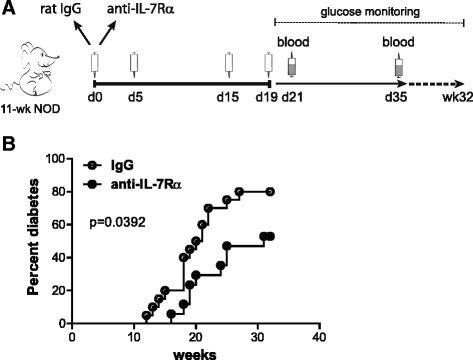
A short-term treatment with anti-IL-7Rα antibodies delays and prevents diabetes in NOD mice. **a** Experimental design outline: 11-week old prediabetic female NOD mice received four doses of 0.5 mg anti-IL-7Rα (*n* = 10) or rat IgG (*n* = 10) antibodies on days 0, 5, 15 and 19. Blood was drawn at day 21 and 35 and T cell populations were analyzed by flow cytometry, and, **b** diabetes incidence was followed in mice that received anti-IL-7Rα antibodies (*black circles*) or rat IgG (*white circles*) measuring urine and blood glucose levels until 32 weeks of age. Data were pooled from two independent experiments (*n* = 20). *P* value is indicated in the figure

We and others previously demonstrated that sustained, long-term treatment (14 weeks) of NOD mice with anti-IL-7Rα monoclonal antibodies (mAbs) robustly prevented diabetes incidence [4, 5]. However, such long-term treatment was accompanied by significant depletion of lymphocyte populations over time, raising concerns about broad immunosuppression and questioning the potential of translating this preventive strategy to the clinic. Therefore, we asked whether a treatment of limited duration with anti-IL-7Rα mAbs would retain benefit for long-term prevention of type 1 diabetes. Treatment was started at 11 weeks of age, when insulitis is known to be evident in NOD mice [19], hence this cohort represented a model of secondary prevention (Fig. 1a). Four doses (0.5 mg each) of anti-IL-7Rα mAbs or control rat IgG were administered intraperitoneally on days 0, 5, 15 and 19. Blood was drawn 2 and 16 days after the last dose was given to evaluate anti-IL-7Rα-induced changes in blood T lymphocytes. In the group that received rat IgG, mice started to become hyperglycemic at 12 weeks of age, whereas the mice treated with anti-IL-7Rα mAbs showed a significant delay in diabetes onset (Fig. 1b). Overall incidence was reduced from ~80% to ~60%, suggesting that in some cases secondary prevention can be achieved with temporary IL-7Rα blockade in prediabetic mice. Thus, a short treatment with blocking antibodies for IL-7Rα retains efficacy to delay and, in some cases, prevent diabetes progression, supporting continued efforts to translate anti-IL-7Rα antibodies for the prevention and treatment of type 1 diabetes.

### Circulating T lymphocytes from anti-IL-7Rα-treated NOD mice show enhanced expression of multiple co-inhibitory receptors

**Fig. 2:**
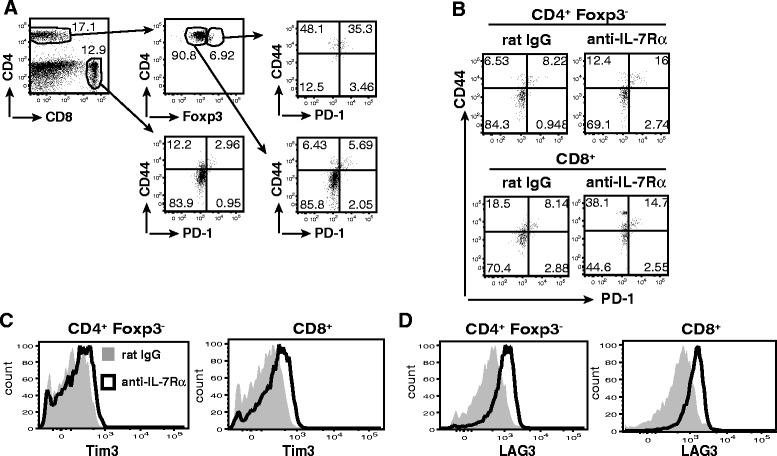
Flow cytometric analysis of peripheral blood T lymphocytes from anti-IL-7Rα − and rat IgG-treated NOD mice. **a** Gating strategy for T_E/M_ cell and Foxp3^+^ Treg populations. **b** Representative dot plots show PD-1 and CD44 percentages within the CD4 + Foxp3- and CD8+ T_E/M_ cell populations. **c**, **d** Representative histogram overlays of Tim-3 and LAG-3 expression within the CD4+ Foxp3- and CD8+ T_E/M_ cell populations

**Fig. 3:**
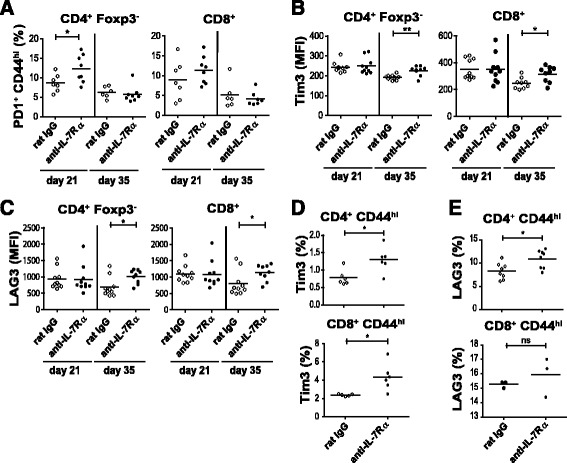
IL-7Rα blockade induces multiple co-inhibitory receptors in CD4^+^ and CD8^+^ T_E/M_ cells. **a**, **b**, **c** Prediabetic female NOD mice received anti-IL-7Rα (*black circles*; *n* = 10) or rat IgG (*white circles*; *n* = 10) antibodies as in Fig. 1a and blood was drawn and analyzed as in Figs. 1a and 2. Percentage of PD-1^+^CD44^high^ cells and MFI of extracellular Tim-3 and LAG-3 within the peripheral blood CD4^+^Foxp3^−^ (*left*) and CD8^+^ (*right*) T_E/M_ cell populations were determined by flow cytometry. All data are representative for two independent experiments. Each symbol represents one individual mouse. * = *p* ≤ 0.05, ** = *p* ≤ 0.005. **d**, **e** Frequency of Tim-3^+^ and LAG-3^+^ cells within the activated (CD44^high^) CD4^+^Foxp3^−^ and CD8^+^ T_E/M_ cell populations in the PLN of mice treated twice per week for two consecutive weeks with anti-IL-7Rα (*black circles*) or rat IgG (*white circles*) antibodies. Data are pooled from two or three independent experiments (*n* = 3–8). * = *p* ≤ 0.05, ns = *p* > 0.05

To gain insight in the changes that occur in T cell phenotypes after short-term IL-7Rα blockade, we performed a flow-cytometric analysis of peripheral blood T cell subsets from treated mice two (day 21) and sixteen (day 35) days after the final antibody administration (Fig. 2). Our previous studies showed that increased expression of the co-inhibitory receptor PD-1 in CD4^+^ T_E/M_ cells from anti-IL-7Rα-treated NOD mice contributed to protection from type 1 diabetes [4]. However, anti-PD-L1 antibodies did not efficiently restore diabetogenicity in CD4^+^ T_E/M_ cells isolated from anti-IL-7Rα-treated NOD mice, suggesting that additional protective mechanisms are induced by IL-7Rα blockade [4]. Therefore, in addition to PD-1, we analyzed the expression of the co-inhibitory receptors LAG-3 and Tim-3, which are known to play important roles in limiting autoimmunity [12, 20, 21], in CD4^+^ and CD8^+^ T cells (Fig. 2). Confirming our previous observations in lymphoid organs [4], the percentage of PD-1^+^ CD44^high^ cells within CD4^+^Foxp3^−^ T_E/M_ cells and CD44^high^CD8^+^ T_E/M_ cells was elevated 2 days after the end of the treatment (day 21) with anti-IL-7Rα vs. rat IgG antibodies (Fig. 3a). Interestingly, these differences were not maintained 2 weeks after the end of treatment. To the contrary, Tim-3 and LAG-3 expression in peripheral blood CD4^+^ and CD8+ T_E/M_ cells was not increased when measured immediately after treatment but was significantly increased 2 weeks after terminating anti-IL-7Rα mAb administration (Fig. 3b, c). Using a separate set of experimental mice, we analyzed the expression of Tim-3 and LAG-3 in the pancreatic lymph nodes (PLN), since this is the site where autoreactive T cells initially respond to β-cell antigens [22]. We found that the frequency of Tim-3^+^ and LAG-3^+^ cells within the CD4^+^ and CD8^+^ T_E/M_ population increased after anti-IL-7Rα mAb treatment (Fig. 3d, e), thus showing that in the PLN these co-inhibitory receptors behave similarly as PD-1, whose expression in PLN was analyzed previously [4]. Taken together, these data show that T lymphocytes in anti-IL7Rα-treated NOD mice exhibit a phenotype reminiscent of T cell exhaustion, characterized by expression of multiple co-inhibitory receptors [23]. Importantly, this phenotype is not only present in the PLN but can be detected in peripheral blood samples, suggesting this could be developed as a biomarker for anti-IL-7Rα treatment. It is intriguing that the kinetics of detecting Tim-3- and LAG-3-expressing cells in blood differs from PD-1, suggesting that the former cells might be retained longer in the PLN or induction of these receptors is delayed.

### Peripheral blood Tregs show increased frequency and an activated phenotype after anti-IL-7Rα antibody treatment

**Fig. 4:**
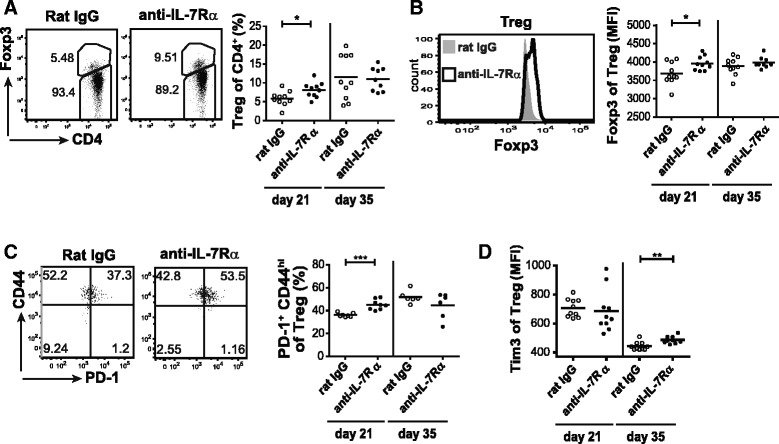
Inhibition of IL-7/IL-7Rα signaling promotes Tregs in peripheral blood. NOD mice were treated and peripheral blood analyzed as in Figs. 1a and 2. **a** Representative dot plots (*left*) and summary (*right*) of frequency of CD4^+^ Foxp3^+^ Tregs within the CD4^+^ T cell population. **b** Representative histogram (*left*) and summary of MFI (*right*) of Foxp3 expression levels within CD4^+^ Foxp3^+^ Tregs. **c**, **d** Representative dot plots (*left*) and summary (*right*) of PD-1^+^ CD44^high^ cells (percentage) and Tim-3 (MFI) within the CD4^+^ Foxp3^+^ Treg population. Each symbol represents one individual mouse. All data are representative for two independent experiments. * = *p* ≤ 0.05, ** = *p* ≤ 0.005, *** = *p* ≤ 0.0005

Tregs play a critical role in the prevention of autoimmunity and deficiencies in their frequency and function are therefore thought to contribute to the development of autoimmune diseases such as type 1 diabetes [15, 16]. In support of this, a lower frequency of Tregs has been detected in the blood of young diabetic patients [24], and it has been described that Tregs from type 1 diabetes patients and NOD mice show functional deficits, possibly related to defects in the IL-2/IL-2Rα pathway [25, 26]. To evaluate whether the frequency and phenotype of circulating Tregs was altered in NOD mice after a short treatment with anti-IL-7Rα mAbs, we analyzed peripheral blood Tregs 2 days (day 21) and 2 weeks (day 35) after final anti-IL-7Rα mAb or rat IgG administration (Fig. 2). The percentage of peripheral blood Foxp3^+^ Tregs was significantly increased 2 days after the end of treatment with anti-IL-7Rα mAbs (Fig. 4a). Importantly, we found that Tregs in peripheral blood of anti-IL-7Rα-treated mice showed increased expression of Foxp3 (Fig. 4b). Increased Foxp3 expression on a per cell basis has been associated with Treg activation and improved suppressive activity [27]. PD-1 expression was also increased in activated (CD44^high^) Tregs from anti-IL-7Rα-treated mice (Fig. 4c), further supporting the notion that IL-7Rα blockade leads to Treg activation [28]. By 2 weeks after the end of the treatment, Treg frequencies, Foxp3 expression and PD-1 had returned to the same levels as controls. However, a significant increase in Tim-3 expression level was observed at this time, hence showing similar expression kinetics as in T_E/M_ cells (Fig. 4d). LAG-3 also showed a trend towards higher expression on day 35 (data not shown). Of note, higher levels of Tim-3 and LAG-3 in Tregs have been associated with an increased suppressive activity of this population [29–31]. Thus, our data indicate that short-term, systemic blockade of IL-7/IL-7Rα signaling not only detectably alters the balance of Tregs/T_E/M_ in peripheral blood but changes Tregs qualitatively by increasing Foxp3 expression and co-inhibitory receptors, suggesting a more activated, suppressive state.

### Co-inhibitory receptor expression on T cells from anti-IL-7Rα-treated mice impairs cytokine production

**Fig. 5:**
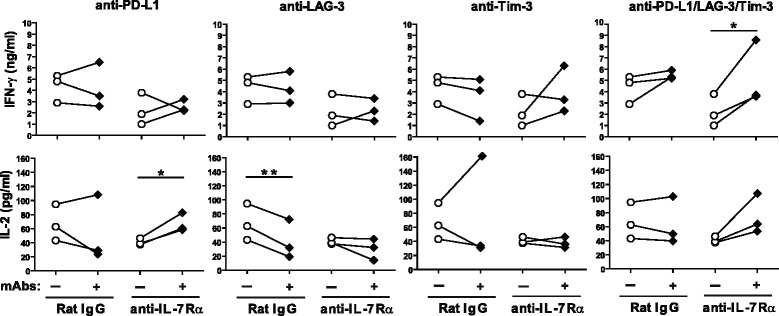
Co-inhibitory receptors reduce cytokine production in T cells from anti-IL-7Rα-treated mice. Female, prediabetic NOD mice (11 weeks old) were treated with anti-IL-7Rα or rat IgG antibodies twice a week for 2 weeks. At the end of treatment, spleen and PLN were harvested and pooled cells stimulated in vitro with anti-CD3 and anti-CD28 antibodies, in the absence (*white circles*) or presence of blocking antibodies against PD-L1, LAG-3 and Tim-3 (*black diamonds*). Supernatants were harvested after 18 h and IFN-γ (*top panels*) and IL-2 (*bottom panels*) levels determined by ELISA. Each symbol represents one individual mouse (*n* = 3). * = *p* ≤ 0.05, ** = *p* ≤ 0.005

To analyze whether co-inhibitory receptors functionally affected T cells from anti-IL-7Rα-treated mice, spleen and pancreatic lymph node cells were stimulated in vitro with anti-CD3 and anti-CD28 antibodies in the presence or absence of PD-L1-, Tim-3- and LAG-3-blocking antibodies (Fig. 5), and IFN-γ and IL-2 production measured. We found that anti-IL-7Rα-treated T cells produced less IFN-γ than controls, consistent with a less functional state of the T cell population. Interestingly, we found that blocking individual co-inhibitory receptors did not efficiently restore IFN-γ production, but blocking PD-L1, Tim-3 and LAG-3 simultaneously significantly increased IFN-γ secretion (Fig. 5). IL-2 production was not reduced in anti-IL-7Rα-treated T cells compared to controls but blocking PD-L1 did result in enhanced secretion. These data demonstrate that expression of co-inhibitory receptors on T cells from anti-IL-7Rα-treated mice impacts their functionality.

### Absence of IL-7 signaling sensitizes T cells to express co-inhibitory receptors in response to TCR stimulation

**Fig. 6:**
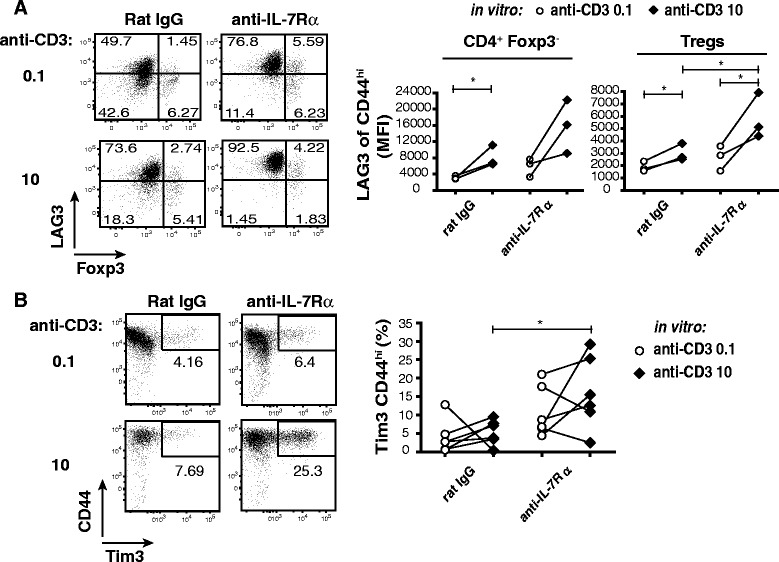
T cells from anti-IL-7Rα-treated mice are sensitized for TCR-triggered co-inhibitory receptor induction. Female, prediabetic NOD mice (10–11 weeks) were treated with anti-IL-7Rα or rat IgG antibodies twice a week for 2 weeks. At the end of treatment, PLN were harvested and cells stimulated *in vitro* with anti-CD3 and anti-CD28 antibodies. **a** Representative dot plots (*left*) show percentages of LAG-3- and Foxp3-expressing cells within the CD4^+^ T cell gate 72 h after stimulation; summary (*right*) of LAG-3 expression levels (MFI) in the indicated cell populations stimulated with 0.1 μg/ml (white circles) or 10 μg/ml (*black diamonds*) anti-CD3. **b** Representative dot plots (*left*) and summary graph (*right*) show percentages of Tim-3^+^ CD44^high^ cells within the CD8^+^ T population, stimulated as indicated. Each symbol represents an individual mouse (*n* = 3–6). Data are representative for two independent experiments. * = *p* ≤ 0.05

Co-inhibitory receptor expression is induced after T cell activation and maintained in situations of chronic antigen stimulation [6–8]. To ask how co-inhibitory receptor expression behaves in response to TCR stimulation in the absence of IL-7/IL-7Rα signals in vivo, we stimulated T cells from the PLN of anti-IL-7Rα or Rat IgG-treated NOD mice in vitro with a low (0.1 μg/ml) or high (10 μg/ml) dose of anti-CD3 mAbs in the presence of anti-CD28 (1 μg/ml) and evaluated LAG-3 and Tim-3 expression. We found that increasing TCR triggering led to higher LAG-3 expression levels in activated (CD44^high^) CD4^+^Foxp3^−^ T_E/M_ cells and Foxp3^+^ Tregs from anti-IL-7Rα-treated mice vs rat IgG controls (Fig. 6a). CD8^+^ T cells did not show increased LAG-3 expression (data not shown). Conversely, Tim-3 expression was increased after strong TCR stimulation of IL-7-deprived CD8^+^ T cells (Fig. 6b) but remained unaffected in CD4^+^ T cells (data not shown), albeit with significant variability between animals. These data demonstrate that T cells persisting in the absence of IL-7 signals become more sensitive towards TCR-induced co-inhibitory receptor expression, further underscoring the idea that IL-7 protects activated T cells from various inhibitory signals, thus promoting the autoimmune response.

## Discussion

In this study we sought to further characterize the protective mechanisms induced by treatment with anti-IL-7Rα antibodies during an ongoing autoreactive T cell response. We found that, in addition to PD-1, two other co-inhibitory receptors, Tim-3 and LAG-3, show increased expression in T cells from anti-IL-7Rα-treated NOD mice. Moreover, IL-7Rα blockade promoted an expansion of the polyclonal Treg population in NOD mice and, interestingly, increased their activation status, indicating enhanced suppressive potential. Our results indicate that a broad program of immunoregulation underlies the slower disease kinetics afforded by IL-7Rα blockade in type 1 diabetes and, that markers of anti-IL-7Rα antibody activity can be detected in peripheral blood following a short course of treatment.

PD-1, Tim-3 and LAG-3 are co-inhibitory receptors that are critical for controlling autoimmunity: studies with blocking antibodies as well as gene-deficient mice demonstrated that these pathways, individually or synergistically, play important roles in type 1 diabetes and other autoimmune diseases [9, 11–13, 32]. Hence, developing methods to promote activity of these pathways is a promising approach towards novel therapies for type 1 diabetes and other autoimmune diseases. Our data indicate that this desired effect is achieved as a consequence of IL-7Rα blockade. Interestingly, such a broad increase of multiple co-inhibitory receptors in CD4^+^ Foxp3^−^ and CD8^+^ T_E/M_ cells resembles the phenotype described in previous studies for exhausted T cells responding to tumors or chronic viral infections [7, 8, 23]. In these settings, continuous antigen exposure results in inhibited, “exhausted” T cells that have lost effector functions (e.g., IFN-γ production) necessary to effectively combat tumors and chronic viral infections [8, 23, 33]. Importantly, IL-7 restored functionality in CD8^+^ T cells during chronic viral infections and in tumor models [34, 35]. These observations thus support the idea that IL-7 is an environmental factor promoting T cell responses during chronic antigen challenge. As a corollary, inappropriate IL-7 signaling during autoreactive T cell activation may contribute to the development of a pathogenic T cell response. In this respect, murine models of autoimmune diseases treated with inhibitors of IL-7/IL-7Rα signaling show preventive or therapeutic efficacy [4, 5, 36–38]. In many of these models, it remains to be investigated whether increased co-inhibitory receptor expression plays a role as a protective mechanism. Due to the multiple immunoregulatory pathways induced in anti-IL-7Rα-treated NOD mice, it will be a challenging endeavor to unequivocally demonstrate the contribution of each to controlling autoimmunity. In this regard, one interesting finding from our study is that cells expressing PD-1 vs Tim-3 and/or LAG-3 appear with different kinetics in the blood after anti-IL-7Rα mAb administration. This may be due to a slower kinetics of initial induction or because Tim-3 and LAG-3-expressing cells are preferentially retained in the lymphoid organs while PD-1 expressing cells belong to a population that more readily enters the circulation. Besides enhanced co-inhibitory receptor expression on T_E/M_ cells, PD-1, Tim-3 and LAG-3 were also increased on Tregs. Tregs expressing PD-1 [39], Tim-3 [31] and LAG-3 [30] are found at sites of active immune responses, e.g., in tumors and transplants, and are thought to possess higher suppressive activity.

The molecular mechanisms underlying increase of co-inhibitory receptors in the absence of IL-7 signaling remain to be determined. However, it is reasonable to speculate that a direct effect of IL-7 on T cells during priming is involved. For example, recent data show that IL-7 provides additional early signals (increased ERK, STAT5, Akt) during TCR engagement that promote optimal T cell activation [40, 41]. These IL-7-induced signals are important for expression of the glucose transporter Glut1 in T cells [42] and, intriguingly, decreased Glut1 and glucose uptake have been associated with increased PD-1 and Tim-3 expression and T cell exhaustion [43]. Hence it is feasible that absence of IL-7 signaling during T cell priming promotes expression of co-inhibitory receptors, perhaps as a consequence of defective metabolic regulation.

## Conclusions

Efforts to translate pre-clinical studies showing efficacy of anti-IL-7Rα antibodies for the treatment of type 1 diabetes have been initiated in the clinic [18]. Our study shows that a limited treatment with anti-IL-7Rα antibodies is sufficient to induce detectable changes in the peripheral blood T cell phenotype, increasing expression of several co-inhibitory receptors in CD4^+^ and CD8^+^ T_E/M_ cells and promoting Treg presence. Hence, our data support the rationale for clinical trials with anti-IL-7Rα mAbs and suggest that a T cell biomarker in the blood based on co-inhibitory receptor expression may be helpful in following individual patients’ response to IL-7Rα blockade. The shorter, 3-week course of treatment we tested here lost some efficacy to prevent type 1 diabetes compared to persistent treatment [4], suggesting that it may be ideally suited to combine with another intervention to improve efficacy while maintaining the increased safety profile presumably associated with limited treatment duration.

## Abbreviations

IL-7: Interleukin-7
mAbs: monoclonal antibodies
NOD: Non-obese diabetic
PLN: Pancreatic lymph nodes
